# Trauma Induces Interleukin-17A Expression on Th17 Cells and CD4+ Regulatory T Cells as Well as Platelet Dysfunction

**DOI:** 10.3389/fimmu.2019.02389

**Published:** 2019-10-11

**Authors:** Friederike Hefele, Alexander Ditsch, Niels Krysiak, Charles C. Caldwell, Peter Biberthaler, Martijn van Griensven, Stefan Huber-Wagner, Marc Hanschen

**Affiliations:** ^1^Experimental Trauma Surgery, Klinikum rechts der Isar, Technical University of Munich, Munich, Germany; ^2^Division of Oncology and Hematology (CCM), Medical Department, Charité–Universitätsmedizin Berlin, Berlin, Germany; ^3^Department of Trauma Surgery, Berufsgenossenschaftliche Unfallklinik Murnau, Murnau, Germany; ^4^Division of Research, Department of Surgery, University of Cincinnati College of Medicine, Cincinnati, OH, United States; ^5^Division of Research, Shriners Hospital for Children, Cincinnati, OH, United States; ^6^Department of Trauma Surgery, Klinikum rechts der Isar, Technical University of Munich, Munich, Germany

**Keywords:** T helper 17 cells, CD4+ regulatory T cells, trauma, hemostasis, IL-17A, flow cytometry, thromboelastometry

## Abstract

**Background:** The organism's immune response to trauma is distinctively controlled, its dysregulation leading to severe post-traumatic complications. Platelets, CD4+ regulatory T cells (CD4+ Tregs) and T helper 17 (Th17) cells have been identified to participate in the post-traumatic immune response. Unfortunately, little is known about their exact role and potential interdependency in humans. Aims of this clinical trial were to phenotype the human immune response following injury and to identify risk factors rendering the host more susceptible to trauma induced injury.

**Methods:** This non-interventional prospective clinical trial enrolled patients following multiple trauma, follow up was conducted for 10 days. Peripheral blood CD4+ Tregs and Th17 cells were analyzed using flow cytometry to determine Interleukin 17A (IL-17A) expression. Hemostasis and platelet function were assessed with rotational thromboelastometry (ROTEM^®^). Subgroup analysis was conducted for the factors gender, age, and trauma severity.

**Results and Conclusion:** This is the first clinical trial to phenotype the immune response following trauma, focusing on platelets, and the adaptive immune response. We discovered a novel increased IL-17A expression on Th17 cells and on CD4+ Tregs following trauma and describe the kinetics of the immune response. The IL-17A response on CD4+ Tregs challenges the ascribed role of CD4+ Tregs to be solely counter inflammatory in this setting. Furthermore, despite a rising number of platelets, ROTEM analysis shows post-traumatic platelet dysfunction. Subgroup analysis revealed gender, age, and trauma severity as influencing factors for several of the analyzed parameters.

## Summary

IL-17A expression on CD4+ Tregs and Th17 cells in multiple trauma patients increases during the first 10 days after trauma with no significant changes in cell numbers detectable. Platelets of trauma patients show signs of dysfunction in thromboelastometry despite increasing counts.

## Introduction

Unintentional injuries, especially road traffic injuries, remain to be the leading cause of death in the age group of 15–29 years, outnumbering the fatalities that result from malaria, tuberculosis, and HIV/AIDS combined, according to the Global Health Estimates data of the World Health Organization (WHO) of 2016 (1). Over all age groups, road traffic injuries increased profoundly, ranking 8th in 2016 as compared to ranking 10th in 2000. The injury-associated mortality is commonly categorized into three groups according to the time of occurrence: within minutes or even seconds following trauma, during the first 24 h, and after several days. The first two groups account for ~50% of all deaths (2). The main reasons for this early mortality are the injury itself and or in combination with pre-existing conditions and can therefore not be effectively prevented. The late mortality, on the other hand, is often caused by multiple organ dysfunction syndrome (MODS) or multiple organ failure (MOF), which are results not only of the trauma but predominantly of the patients' dysfunctional immune response (3). The post-traumatic immune responses can be categorized into proinflammatory and anti-inflammatory reactions, commonly referred to as systemic inflammatory response syndrome (SIRS) and compensatory anti-inflammatory response syndrome (CARS) as well as mixed anti-inflammatory response syndrome (MARS) (4). An imbalance between these pro- and anti-inflammatory immune responses is believed to be the cause for heightened susceptibility to infections and organ damage. While trauma itself and the associated early mortality cannot be undone, the late mortality due to the host's immune response following trauma is potentially modifiable. As the mechanisms of the injured bodies' immune system to react to severe trauma are still not fully understood and due to the fact that most knowledge has been gathered in animal models, this study aims to phenotype the human immune response in severely injured patients.

Due to recent findings in the literature, highlighting the role and interplay of CD4+ Tregs, Th17 cells, and platelets following trauma, our study attempts to address these key players. A decreased CD4+ Treg/Th17 cell ratio was recently discovered in a multiple trauma rat model, correlating inversely with disease activity (5). In our previous studies utilizing a murine burn injury model, we have been able to show impaired activation of intracellular pathway signaling in CD4+ Tregs after platelet depletion (6). Hence, we were the first to show reciprocal activation of CD4+ Tregs and platelets following trauma induced injury. The pathways responsible for the modulation of CD4+ Treg activation seem to be tumor necrosis factor receptor 2- and toll-like receptor 4- dependent (7).

Trauma is associated with a suppressed Th1 immune system phenotype and an expansion of CD4+ Tregs (8). Of interest, little is known of Th17 cells in the setting of trauma.

T helper 17 (Th17) cells are a recently discovered new lineage of T helper cells named after their production and expression as well as secretion of IL-17 (9). Their characteristic cytokine profile—IL-17, IL-17F, IL-9, IL-10, IL-21, IL-22, IFN-γ, and GM-CSF—makes them mostly proinflammatory cells (10–15). The transcription factor retinoid acid-related orphan receptor gamma t (RORγt) was revealed to be the key regulator in the development of this T helper cell subset, additionally a similar potential could be proven for retinoid acid-related orphan receptor alpha (RORα) (16, 17). In autoimmunity, the primary research field for Th17 cells, targeting the p40 subunit of IL-23 and IL-12 with Ustekinumab as well as the IL-17-receptor with Brodalumab has proven to be a highly efficient therapeutic option for psoriasis (18, 19). Aside from causing several autoimmune disorders, Th17 cells partake in microbial defense—they are integral in the clearance of extracellular pathogens like *Klebsiella pneumoniae* and *Candida albicans* (20, 21). No specific set of identifying markers has yet been agreed on for this cell type, however, several studies have used CD161 and chemokine receptor 6 (CCR6/CD196) to identify CD4+ lymphocytes as Th17 cells (22–24). In 2009, Brucklacher-Waldert et al. showed IL-17A surface expression identifies Th17 cells and correlates with its intracellular production (25). While Th17 cells have been mainly considered with autoimmune disease in the past, recent findings point out a potential interplay with platelets in the setting of burn and trauma (26). The mechanisms and role of platelet-Th17 interaction following trauma need yet to be characterized.

CD4+ regulatory T cells (CD4+ Tregs) have been established key players in the post-traumatic immune response, they contribute to the counterinflammatory reaction to severe injury (27). CD4+ Tregs were first described by Sakaguchi et al. as suppressors of T effector cell activation and proliferation in 1995 and were characterized as highly expressing CD25 (IL-2-receptor α chain) (28). Several other markers for this cell type have since been identified, the most commonly used being intracellular expression of transcription factor forkhead box p3 (Foxp3) and lack of surface-CD127-expression (29, 30). CD4+ Tregs play a crucial role in maintaining immunologic self-tolerance and preventing excessive immune reactions to weak stimuli, preserving the delicate, and crucial balance between pro- and anti-inflammatory immune reactions (31, 32). However, they also display a significant potential for plasticity and adaptability: in certain settings, CD4+ Tregs can convert into Th17 cells (33). Furthermore, in 2009, a subset of IL-17-producing CD4+ Tregs was discovered by Voo et al. (34).

Platelets, for a long time only recognized for their pivotal role in the coagulation system, are nowadays established as a key component of the immune system (35–37). By releasing cytokines and chemokines from their granules, they participate as mediators in host defense against pathogens. However, platelets also act as effector cells of the immune system by releasing bactericidal defensins (38). Platelets can even synthesize new molecules as they contain mRNA; furthermore, recent findings suggest that the type of mRNA they are equipped with varies depending on the state of the host—healthy or sick (39). As pointed out above, platelets have the capability to modulate the immune response following injury. The interaction with Th17 cell and CD4+ Tregs as well as its role following injury need yet to be characterized, studies in humans are mostly missing.

While animal models are necessary and crucial to elucidate new aspects of basic immunological pathomechanisms, there are considerable interspecies differences (40). In order to discover new therapeutic targets to combat the post-traumatic immune dysfunction, a more complete knowledge of the key players and mechanisms involved in the human post-traumatic response is integral.

Taken together, we conducted a clinical prospective non-interventional trial on patients following multiple trauma, utilizing serial blood analyses. The aims of this study were first to phenotype the post-traumatic immune response focusing on lymphocytes (Th17 cells, CD4+ Tregs) and platelets, and second to investigate the host's susceptibility for trauma by conducting subgroup analysis. We used flow cytometry and rotational thromboelastometry (ROTEM^®^) to characterize the cells and their functionality.

We discovered a significant increase in IL-17A expression on both Th17 cells and CD4+ Tregs during the first 10 days after trauma. Our findings challenge the ascribed role of CD4+ Tregs to be solely counterinflammatory in the setting of trauma induced injury. In our thromboelastometric measurements we found an increase in maximum clot firmness (MCF) alongside with post-traumatic platelet dysfunction. Furthermore, assessment of gender, age, and trauma severity measured by the injury severity score (ISS) as possible influencing factors yielded significant differences in several of the analyzed parameters. The presented subgroup analysis supports the concept, that the immune response following trauma is shaped according to the susceptibility of the host.

## Materials and Methods

### Patients

For our prospective non-interventional study, we recruited severely injured patients from ages 18 to 95 with multiple traumata defined by an injury severity score (ISS) ≥16. Exclusion criteria were pregnancy and imprisonment. The patients included in the study were brought to the emergency room of the Klinikum rechts der Isar of the Technical University of Munich no more than 12 h after their respective trauma between December 2014 and March 2017. Written informed consent was obtained from all patients or their relatives according to the patient's suspected will. The study follows the principles of the declaration of Helsinki with its novelizations of Tokyo 1975, Hongkong 1989, and Somerset West 1996. It was approved by the ethics review committee of the Technical University of Munich (reference number 5925/13) prior to starting the research.

### Reagents

For flow cytometry, cells were stained in PBA buffer: PBS supplemented with bovine serum albumin and sodium azide (all by Sigma Aldrich, St. Louis, MO). For red blood cell lysis, we used Schwinzer solution: 1 l distilled water supplemented with 8.3 grams of ammonium chloride (Carl Roth GmbH + Co. KG, Karlsruhe, Germany), 1.0 g of potassium carbonate (Caesar & Lorentz GmbH, Hilden, Germany) and 0.1 g of ethylenediaminetetraacetic acid (EDTA) (Carl Roth GmbH + Co. KG, Karlsruhe, Germany). Fc-blocking agent (eBioscience, San Diego, CA) was used to prevent non-specific binding of staining antibodies. Surface staining was performed using allophycocyanin (APC)-labeled anti-CD4 (eBioscience, San Diego, CA), eFluor450™-labeled anti-CD161 (eBioscience, San Diego, CA), FITC-labeled anti-CD196 (eBioscience, San Diego, CA), FITC-labeled anti-CD4 (eBioscience, San Diego, CA), eFluor450™-labeled anti-CD25 (eBioscience, San Diego, CA), APC-Cy7-labeled anti-CD127 (eBioscience, San Diego, CA) and phycoerythrin (PE)-labeled anti-IL17A (LifeSpan BioSciences, Seattle, WA). MACSQuant^®^ calibration beads (Miltenyi Biotec GmbH, Bergisch Gladbach, Germany) were used prior to measurements according to the manufacturer's instructions.

For thromboelastometric analysis we used star-tem, r ex-tem, in-tem, and fib-tem reagents (all by TEM International GmbH, Munich, Germany).

### Sample and Data Retrieval

After hospital admission and inclusion of the patient in the study according to the criteria stated above, a series of nine blood draws was performed at several time points after trauma: the first one directly in the emergency room, next after 6, 12, 24, 48, and 72 h, furthermore after 5, 7, and 10 days. At each time point we collected citrated whole blood for thromboelastometric analysis of platelets and EDTA-treated whole blood for flow cytometrical lymphocyte analysis. Sarstedt S-Monovette^®^ 3 ml with citrate 3,2% (1:10) and Sarstedt S-Monovette^®^ 9 ml with K3 EDTA (both Sarstedt AG & Co. KG, Nümbrecht, Germany) were used for blood collection.

Furthermore, absolute platelet counts and demographic as well as clinical patient data (demographics, injury mechanism, and severity) were gathered.

### Flow Cytometry

Flow cytometric analysis of T-cell subpopulations was performed on a MACSQuant^®^ device (Miltenyi Biotec GmbH, Bergisch Gladbach, Germany). Samples were prepared as follows: within 15 min after blood draw, EDTA-treated blood was added to Schwinzer red blood cell lysis solution. After 15 min of incubation at 4°C the cells were washed and buffered with PBA and plated on 96-well round bottom plates for staining. Fc-blocking agent was added to prevent unspecific antibody binding.

After incubation we used APC-labeled anti-CD4, eFluor450™-labeled anti-CD161, and FITC-labeled anti-CD196 for detection of Th17 cells. For CD4+ Treg detection we used FITC-labeled anti-CD4, eFluor450™-labeled anti-CD25, and APC-Cy7-labeled anti-CD127. Cell activation was evaluated using PE-labeled anti-IL17A. After incubation, cells were washed and resuspended in PBA buffer for immediate flow cytometric analysis. Prior to measurements, the MACSQuant^®^ was calibrated using MACSQuant^®^ calibration beads according to the manufacturer's instructions.

The obtained data sets were analyzed using FlowJo Software (FlowJo LLC, Ashland, OR). After single cell selection, Th17 cells were defined as CD4+, CD161+, CD196+ cells, and CD4+ Tregs were defined as CD4+, CD25+, CD127^−^ cells. IL-17A expression on Th17 cells was assessed by calculating the relative median fluorescence intensity (MFI) of PE on Th17 cells stained with PE-conjugated anti-IL17A antibody. To calculate the relative MFI, the MFI of PE on anti-IL17A stained cells was divided by the MFI of PE measured on Th17 cells not stained with any PE-conjugated antibody. IL-17A expression on CD4+ Tregs was assessed analogously.

### Thromboelastometry

For thromboelastometric assessment of platelet function we used the ROTEM^®^ delta device (TEM International GmbH, Munich, Germany) according to the manual. We examined platelet function after extrinsic activation of the coagulation cascade using star-tem reagent to recalcify the citrated blood and using r ex-tem reagent to activate platelets. For evaluation of plasmatic coagulation without influence of platelets fib-tem reagent was added to inhibit platelet function before starting the measurement.

We analyzed the parameter maximum clot firmness (MCF), measured in millimeters (mm) as the greatest amplitude of the reaction curve. For evaluation of the platelet contribution of the maximum clot firmness, the platelet MCF was calculated as the MCF (extem-fibtem) by subtracting the MCF measured in fibtem from the one measured in extem, as it has been described in literature before (41).

### Statistics

The Statistical Package for the Social Sciences (SPSS) (IBM, Armonk, NY) was used to perform statistical analysis. In the descriptive statistical analysis values are given as mean ± standard deviation. Generalized estimating equations (GEE) with an exchangeable correlation matrix were applied to test for statistically significant changes over time, which was measured in days. Gender, dichotomized age (<55 years, ≥55 years), and ISS (<25 or ≥25) were calculated in as influencing factors. Values given are the regression coefficient (B), the standard error (SE), the 95% confidence interval (95% CI) and the probability value (*p*). *p* < 0.05 was considered significant.

## Results

### Study Population

Twenty trauma patients were enrolled in the study, the detailed epidemiologic data are provided in Table 1. Of the included patients, 70% (14) were male. The mean patient age was 46.5 ± 18.7 years, in 65% (13) of all cases, the injuries were caused by road traffic accidents. The mean ISS was 28.4 ± 11.8, 55% (11) of the study cohort had an ISS ≥25. Injuries to the extremities including the pelvic girdle were the most common, affecting 85% (17) of the patients, followed by injuries to the chest, which affected 80% (16) of the study cohort. With a mean abbreviated injury scale (AIS) of 3.7 ± 1.0, injuries to the chest also were the most severe. 85% (17) of the patients survived. In the survivor group, the mean intensive care unit stay was 13.2 ± 16.6 days and 3.8 ± 2.0 surgical procedures were performed.

**Table 1:** Demographic patient data.

**Demographic**	**Frequency (percentage)**	**Mean ± SD**
Number of patients (*n*)	20 (100%)	
Age [years]		46.5 ± 18.7
<55	13 (65%)	36.0 ± 13.5
≥55	7 (35%)	66.0 ± 7.7
Gender		
Male	14 (70%)	
Female	6 (30%)	
Injury mechanism		
Road traffic accident	13 (65%)	
Suicide attempt	4 (20%)	
Other	3 (15%)	
AIS scores		
Head/neck	13 (65%)	2.6 ± 1.2
Face	9 (45%)	2.8 ± 0.7
Chest	16 (80%)	3.7 ± 1.0
Abdomen/pelvis contents	6 (30%)	2.5 ± 1.0
Extremities/pelvic girdle	17 (85%)	2.9 ± 0.8
External	10 (50%)	1.1 ± 0.3
ISS		28.4 ± 11.8
<25	9 (45%)	18.3 ± 1.7
≥25	11 (55%)	36.6 ± 9.8
Non-survivors	3 (15%)	
Survivors	17 (85%)	
Length of ICU stay [days]		13.2 ± 16.6
Number of surgeries performed		3.8 ± 2.0

*SD, standard deviation; AIS, abbreviated injury scale; ISS, injury severity score; ICU, intensive care unit*.

### Th17/CD4+ Ratio

**Figure 1:**
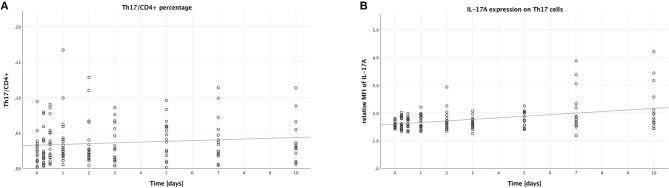
Percentage of Th17 cells in CD4+ lymphocytes and IL-17A expression on Th17 cells in peripheral blood of multiple trauma patients, assessed via flow cytometry. Blood was drawn at nine time points: in the emergency room (0), after 6 and 12 h, and after 1, 2, 3, 5, 7, and 10 days. Each dot displays a single patient and time point. The fit lines are for illustrative purposes and do not represent the generalized estimating equation (GEE). **(A)** Th17 cells were defined as CD4+, CD161+, CD196+. There was no significant increase in the percentage of Th17 cells in CD4+ lymphocytes during the first 10 days after trauma (B = 0.001, SE = 0.001, 95% CI [0.000; 0.002], *p* = 0.094). **(B)** IL-17A expression on Th17 cells was assessed by calculating the relative median fluorescence intensity (MFI) of PE on Th17 cells stained with PE-conjugated anti-IL-17A antibody, dividing the MFI of PE on anti-IL-17A-stained Th17 cells by the MFI of PE on Th17 cells not stained with any PE-conjugated antibody. During the first 10 days after trauma, there was a significant increase in the IL-17A expression on Th17 cells (B = 0.056, SE = 0.017, 95% CI [0.022; 0.090], *p* = 0.001).

To assess the number of Th17 cells, we looked into the percentage of Th17 cells (CD4+, CD161+, CD196+) to all CD4+ lymphocytes. This percentage did not change significantly over time (B = 0.001, SE = 0.001, 95% CI [0.000; 0.002], *p* = 0.094), which is also displayed in Figure 1A. However, males had higher Th17/CD4+ ratios than females (B = 0.021, SE = 0.009, 95% CI [0.003, 0.039], *p* = 0.024), while younger (<55 years of age) patients had lower ratios when compared to older (≥55 years of age) patients (B = −0.017, SE = 0.007, 95% CI [−0.031,−0.003], *p* = 0.017). No significant difference was visible when comparing lower (<25) to higher ISS (≥25) (B = −0.003, SE = 0.119, 95% CI [−0.026, 0.021], *p* = 0.825).

### IL-17A Expression on Th17 Cells

Next, we analyzed the median fluorescence intensity of the PE-conjugated IL-17A-antibody on Th17 cells to measure the IL-17A expression of these cells. As shown in Figure 1B, the IL-17A expression on Th17 cells increased over time (B = 0.056, SE = 0.017, 95% CI [0.022; 0.090], *p* = 0.001). However, no significant differences were seen when comparing the subgroups: males to females (B = −0.053, SE = 0.150, 95% CI [−0.346; 0.241], *p* = 0.725), younger to older patients (B = −0.011, SE = 0.167, 95% CI [−0.338; 0.316], *p* = 0.946), and lower to higher ISS (B = −0.002, SE = 0.101, 95% CI [−0.200; 0.197], *p* = 0.987).

### CD4+ Treg/CD4+ Ratio

**Figure 2:**
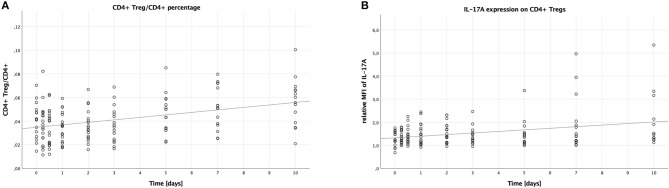
Percentage of CD4+ Tregs in CD4+ lymphocytes and IL-17A expression on CD4+ Tregs in peripheral blood of multiple trauma patients, assessed via flow cytometry. Blood was drawn at nine time points: in the emergency room (0), after 6 and 12 h, and after 1, 2, 3, 5, 7, and 10 days. Each dot displays a single patient and time point. The fit lines are for illustrative purposes and do not represent the generalized estimating equation (GEE). **(A)** CD4+ Tregs were defined as CD4+, CD25+, CD127^−^. The CD4+ Treg/CD4+ lymphocytes percentage increased significantly during the first 10 days after trauma (B = 0.002, SE < 0.001, 95% CI [0.001; 0.003], *p* < 0.001). **(B)** IL-17A expression on CD4+ Tregs was assessed by calculating the relative median fluorescence intensity (MFI) of PE on CD4+ Tregs stained with PE-conjugated anti-IL-17A antibody, dividing the MFI of PE on anti-IL-17A stained CD4+ Tregs by the MFI of PE on CD4+ Tregs not stained with any PE-conjugated antibody. There was a significant increase in the IL-17A expression on CD4+ Tregs in the peripheral blood of patients during the first 10 days after trauma (B = 0.064, SE = 0.022, 95% CI [0.021; 0.107], *p* = 0.004).

Analogously to the Th17 cells, the percentage of CD4+ Tregs to all CD4+ lymphocytes was calculated to measure the number of CD4+ Tregs. Contrary to the percentage of Th17 cells, the percentage of CD4+ Tregs (CD4+, CD25+, CD127^−^) to all CD4+ lymphocytes increased over time (B = 0.002, SE <0.001, 95% CI [0.001; 0.003], *p* < 0.001), as shown in Figure 2A. Like with the Th17/CD4+ ratio, males had higher CD4+ Treg/CD4+ ratios compared to females (B = 0.008, SE = 0.003, 95% CI [0.003; 0.014], *p* = 0.004), while younger patients had lower ratios compared to older patients (B = −0.016, SE = 0.004, 95% CI [−0.024; −0.009], *p* < 0.001). No significant difference was to be seen when comparing lower to higher ISS (B = −0.007, SE = 0.004, 95% CI [−0.014; >0.000], *p* = 0.056).

### IL-17A Expression on CD4+ Tregs

Again, just like on Th17 cells, we analyzed the median fluorescence intensity of the PE-conjugated IL-17A antibody to assess the IL-17A expression on CD4+ Tregs. This expression increased over time (B = 0.064, SE = 0.022, 95% CI [0.021; 0.107], *p* = 0.004), the according scatter plot is shown in Figure 2B. Again, like with the IL-17A expression on Th17 cells, no significant differences were detectable when comparing the subgroups: males to females (B = −0.170, SE = 0.309, 95% CI [-0.777; 0.436], *p* = 0.582), younger to older patients (B = −0.222, SE = 0.311, 95% CI [−0.832; 0.387], *p* = 0.475), and lower to higher ISS (B = 0.108, SE = 0.195, 95% CI [−0.276; 0.491], *p* = 0.582).

### Absolute Platelet Count

**Figure 3:**
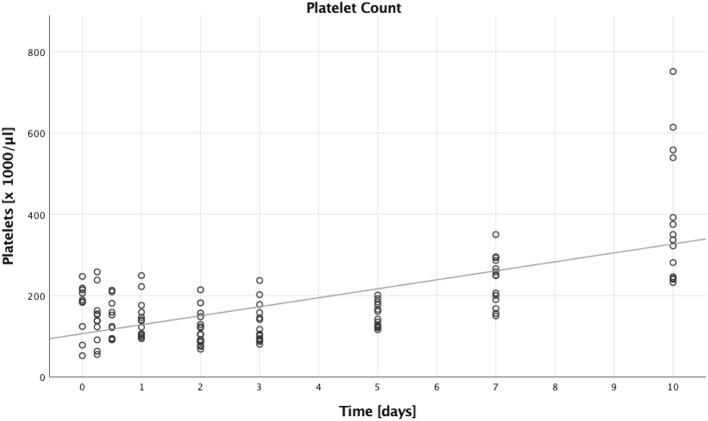
Absolute platelet count. Absolute platelet counts of multiple trauma patients were assessed on nine time points: in the emergency room (0), after 6 and 12 h, and after 1, 2, 3, 5, 7, and 10 days after trauma. Each dot displays a single patient and time point. The fit line is for illustrative purposes and does not represent the generalized estimating equation (GEE). A significant increase in absolute platelet counts could be seen during the first 10 days after trauma (B = 22.178, SE = 3.523, 95% CI [15.273; 29.082], *p* < 0.001).

Next, we looked into the number of platelets, which increased over time (B = 22.178, SE = 3.523, 95% CI [15.273; 29.082], *p* < 0.001). This is illustrated in Figure 3, furthermore we saw higher platelet counts in patients with lower ISS compared to patients with higher ISS (B = 63.530, SE = 28.279, 95% CI [8.104; 118.955], *p* = 0.025). There were, however, no significant differences when comparing males to females (B = 32.740, SE = 26.570, 95% CI [−19.337; 84.817], *p* = 0.218), and younger to older patients (B = 11.333, SE = 16.680, 95% CI [−21.360; 44.026], *p* = 0.497).

### MCF in Extem Measurements

**Figure 4:**
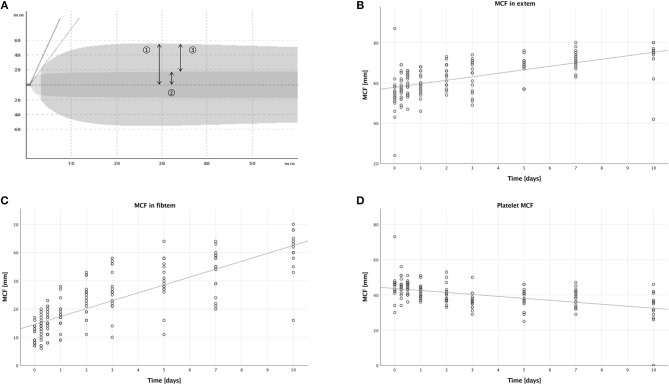
Thromboelastometric assessment of hemostasis after multiple trauma using the ROTEM^®^ device. Blood was drawn at nine time points after trauma: in the emergency room (0), after 6 and 12 h, and after 1, 2, 3, 5, 7, and 10 days. Each dot displays one measurement. The fit lines are for illustrative purposes and do not represent the generalized estimating equation (GEE). **(A)** An overlay of two typical graphic displays of ROTEM^®^ measurements, so-called TEMograms, is shown: The outer curve is the result of an extem measurement, which imitates the extrinsic activation of the coagulation cascade. The inner curve is the result of a fibtem measurement, in which platelets are inhibited and therefore only the plasmatic part of the coagulation is assessed. The parameter maximum clot firmness (MCF) is represented by arrow 1 for the extem measurement and by arrow 2 for the fibtem measurement. Subtracting the fibtem MCF from the extem MCF results in arrow 3, the platelet MCF, which represents the platelet contribution to the overall MCF. **(B)** The MCF measured with the extem panel increased significantly during the first 10 days after trauma (B = 1.677, SE = 0.319, 95% CI [1.053; 2.301], *p* < 0.001). **(C)** A significant increase of the MCF measured with the fibtem panel was also detectable (B = 2.790, SE = 0.151, 95% CI [2.494; 3.087], *p* < 0.001). **(D)** However, the platelet MCF decreased significantly (B = −1.141, SE = 0.296, 95% CI [−1.721;−0.561], *p* < 0.001).

With extem, the patients' hemostatic function after extrinsic activation of the coagulation cascade is measured (compare Figure 4A).

In the extem measurements, the MCF increased over time, as seen in Figure 4B, (B = 1.677, SE = 0.319, 95% CI [1.053; 2.301], *p* < 0.001), and males had higher MCFs compared to females (B = 7.220, SE = 2.366, 95% CI [2.583; 11.858], *p* = 0.002). There were no significant differences in the other subgroups, when comparing younger to older patients (B = −1.877, SE = 1.856, 95% CI [−5.516; 1.761], *p* = 0.312), or lower to higher ISS (B = 1.174, SE = 1.785, 95% CI [−2.324; 4.673], *p* = 0.511).

### MCF in Fibtem Measurements

The fibtem panel allows the assessment of hemostatic capacity without platelets after extrinsic coagulation activation.

In the fibtem measurements, the MCF increased over time as well, which is shown in Figure 4C, (B = 2.790, SE = 0.151, 95% CI [2.494; 3.087], *p* < 0.001) with a higher MCF in males compared to females (B = 3.948, SE = 1.551, 95% CI [0.909; 6.987], *p* = 0.011). We also measured a lower MCF in patients with a lower ISS compared to patients with a higher ISS (B = −3.493, SE = 1.126, 95% CI [−6.034; −0.952], *p* = 0.007). There were no significant differences when comparing younger to older patients (B = 0.346, SE = 1.658, 95% CI [−2.905; 3.596], *p* = 0.835).

### Platelet MCF: MCF(extem)–MCF(Fibtem)

To assess the platelet contribution to the clot firmness, we subtracted the MCF measured in fibtem at each time point from the MCF measured in extem.

Contrary to the MCFs measured in extem and fibtem, we could see a decrease over time in this calculated MCF (B = −1.141, SE = 0.296, 95% CI [−1.721; −0.561], *p* < 0.001), which is illustrated in Figure 4D. Furthermore, younger patients had a lower calculated platelet MCF than older patients (B = −2.633, SE = 0.660, 95% CI [−3.926; −1.339], *p* < 0.001). Corresponding to the findings in the fibtem MCF, patients with a lower ISS had a higher calculated platelet MCF than patients with a higher ISS (B = 3.808, SE = 1.065, 95% CI [1.720; 5.896], *p* < 0.001). No significant differences were seen when comparing males to females (B = 1.776, SE = 1.006, 95% CI [−0.195; 3.746], *p* = 0.077).

### Subgroup Analysis Summary

An overview of the influencing factors is given in Table 2.

**Table 2:** Overview of the subgroup analysis of influencing factors.

**Value**	**Gender (male vs. female)**		**Age (<55 vs**. **≥55 years)**		**ISS (<25 vs**. **≥25)**	
	**B**	***p*-value**	**B**	***p*-value**	**B**	***p*-value**
Th17/CD4+	0.021	0.024	−0.017	0.017		n.s.
IL-17A on Th17		n.s.		n.s.		n.s.
CD4+ Treg/CD4+	0.008	0.004	−0.016	<0.001		n.s.
IL-17A on CD4+ Tregs		n.s.		n.s.		n.s.
Platelet count		n.s.		n.s.	63.530	0.025
MCF extem	7.220	0.002		n.s.		n.s.
MCF fibtem	3.948	0.011		n.s.	−3.493	0.007
Platelet MCF		n.s.	−2.633	<0.001	3.808	<0.001

*Positive regression coefficients (B) mean higher values in the male, younger age, or lower ISS group, respectively, while negative regression coefficients mean lower values in the male, younger age, or lower injury severity score (ISS) group*.

*Th17, T helper 17; CD4+ Treg, CD4+ regulatory T cell; MCF, maximum clot firmness; n.s., not significant*.

Males had significantly higher Th17/CD4+ and CD4+ Treg/CD4+ lymphocytes ratios, furthermore the males' MCF measured in extem and fibtem was significantly higher than the females'.

Age influenced Th17/CD4+ and CD4+ Treg/CD4+ lymphocytes ratios as well: both were significantly lower in younger compared to older patients. Additionally, age had an impact on the platelet MCF, with a significantly lower calculated platelet MCF in younger compared to older patients.

The ISS affected platelets and coagulation: The absolute platelet count was higher in patients with lower ISS as compared to patients with higher ISS. The fibtem MCF was significantly lower and the platelet MCF significantly higher in patients with lower ISS than in patients with higher ISS.

## Discussion

The immune response to trauma is a precisely regulated process with pro- and anti-inflammatory components; their imbalance or derailment can lead to fatal consequences and is associated with a high mortality due to sepsis and/or the development of MOF. While there have been made significant advances in treating the direct impact of trauma to the body, i.e., fractures, soft tissue, and organ injuries, no well-established therapeutic concept for the subsequent immune reaction exists to date. Furthermore, diagnostic measures to assess the immune response of the host following trauma are missing so far. A distinct understanding of the regulating mechanisms as well as the involved cells is crucial for the development of treatment strategies to not only manage immunologic complications, but prevent their occurrence entirely.

In this study, we analyzed the peripheral blood derived from multiple trauma patients at nine specific time points, from admission to the emergency department until 10 days after trauma. This comparatively long observation period allowed us to detect even small alterations in the examined parameters. Furthermore, we were able to describe the development in trauma patients over time, opposed to comparing with a control group. For the characterization of CD4+ Tregs and Th17 cells, we applied flow cytometry, a well-established method for the fast screening of large numbers of cells. Its high-volume output facilitates the analysis of particularly small cell populations like Th17 cells and CD4+ Tregs. The resulting data are quantifiable and highly comparable. For the assessment of hemostasis and platelet function, on the other hand, we chose rotational thromboelastometry (ROTEM^®^), which is applicable for clinical diagnostics as well as research purposes. Designed as a point of care technology device, it is resilient to errors and offers fast and comparable results.

In regards to Th17 cells in trauma patients, this study shows that while the number of these cells in peripheral blood does not increase significantly during the first 10 days after trauma (Figure 1A), their surface expression of IL-17A actually does (Figure 1B). Due to Th17 cells only being discovered recently in 2006 as a new subset of T helper cells, the research connecting this cell type with trauma is quite scarce. In 2013, Dai et al. found a decreased CD4+ Treg/Th17 cell ratio in a multiple trauma rat model compared to sham treatment 4 h after trauma. There were, however, no significant differences in the numbers of Th17 cells, which corresponds with our findings (5). Treating the animals with anti-IL-17A antibody, lung inflammation parameters improved, which points to a potentially harmful role for IL-17A producing Th17 cells after trauma (42). Another study from 2016 on trauma patients found elevated Th17/CD4+ Treg ratios in trauma patients who developed sepsis, furthermore the ratio of Th17 cells to CD4+ Tregs was skewed in favor of Th17 cells in non-surviving patients (43). This is once again in line with the view that Th17 cells might have a rather detrimental effect in a post-traumatic setting. A clinical trial on sepsis patients demonstrated elevated Th17 cell percentages as well as increased Th17/CD4+ Treg ratios in non-survivors with a positive correlation to the acute physiology and chronic health evaluation II (APACHE II) score (44). In another recently published clinical trial on intensive care unit (ICU) patients, elevated serum IL-17 levels have been shown to be a predictor for the development of sepsis, once more supporting the notion of the rather harmful role of Th17 cells (45).

Ever since the discovery of CD4+ Tregs in 1995 by Sakaguchi et al., they have been in the focus of immunologic research (28). In trauma, their role has been suggested to be mostly protective, as they are able to suppress excessive inflammation, therefore potentially inhibiting the development of severe inflammatory response syndrome (SIRS). CD4+ Tregs have been shown to be activated early after injury in a murine burn model (46). Furthermore, also using the murine burn model, we have earlier been able to demonstrate an interaction of CD4+ Tregs with platelets, this interaction being modulated by TNF-RII- and TLR4-dependent pathways (6, 7). In this study, regarding CD4+ Treg count, we saw an increasing number relative to all CD4+ lymphocytes during the observed time period (Figure 2A). However, a study on multiple trauma patients published by Serve et al. in 2018 showed a drop in the CD+ Treg/CD4+ lymphocyte ratio compared to healthy volunteers (47). This data might seem contradictory to our recent findings; however, their observation period was shorter with the last time point at 72 h, furthermore we have not compared our results to healthy volunteers yet.

The established concept of CD4+ Tregs as being solely immunosuppressive cells has recently been challenged, as several studies have shown a considerable amount of plasticity for CD4+ Tregs, especially toward the Th17 lineage (33, 34). Surprisingly, our data show that IL-17A expressing CD4+ Tregs occur after trauma and that their expression of IL-17A even increases during our observed time period (Figure 2B). Until now, CD4+ Tregs have been primarily associated with the compensatory anti-inflammatory response syndrome (CARS) following trauma, yet our data showing proinflammatory cytokine production by CD4+ Tregs in multiple trauma patients certainly necessitate a more differentiated look on these cells.

The detection of an interaction of CD4+ CD4+ Tregs with platelets in a murine burn model led us to investigate platelet function in this study on multiple trauma patients as well (6). Our thromboelastometric results show an increasing MCF both in the extem and fibtem measurements (Figures 4B,C); however, subtracting the fibtem MCF from the extem MCF, thereby calculating the platelet contribution or platelet MCF (41), we actually saw a decrease (Figure 4D). The rise in clot firmness we saw in extem and fibtem might be attributable to the substitution of fibrinogen and clotting factors trauma patients often receive, the decreasing platelet function on the other hand remains to be an interesting topic. The phenomenon of a post-traumatic platelet dysfunction despite a reassuring platelet count has also been described by Kutcher et al., moreover they identified a low Glasgow coma scale (GCS) as an independent predictor (48). A possible cause for the platelet dysfunction could be consumption after activation, in line with the development of disseminated intravascular coagulation, yet our data show an actual rise in numbers during the observed time period with a simultaneous dysfunction (Figures 3, 4D). Therefore, consumption alone does not serve as a sufficient explanation. The platelet count increase might—at least partly—occur due to transfusions, and studies have shown that these transfused cells do forfeit some of their function as a result of platelet storage lesions (49). Lastly, it remains in question, as to what extent this observed dysfunction in hemostasis also concerns the immunologic role of platelets.

To facilitate a more nuanced evaluation of our data, we chose generalized estimating equations for the statistical analysis, as they provide the opportunity of including potential influencing factors. We chose to integrate gender, age, and trauma severity in our calculations, whereby age and trauma severity were dichotomized: “<55 years” and “≥55 years,” “ISS <25” and “ISS ≥ 25.” The summarized results are displayed in Table 2. Conflicting data exists on the frequencies of Th17 cells in older compared to younger healthy individuals: there is evidence for higher (50) as well as for lower numbers in the elderly population (51). Our data shows a higher percentage of Th17 cells in older compared to younger multiple trauma patients. This offers an explanation on a cellular level for the typically worse outcome of elderly patients suffering multiple trauma, considering that higher numbers of Th17 cells and elevated IL-17A levels are generally associated with a higher mortality. In healthy individuals, CD4+ Treg frequencies increase during the aging process; evidence for this has been provided in animal as well as human studies (52, 53). We have now shown that this applies to trauma patients as well: we found elevated CD4+ Treg/CD4+ percentages in older compared to younger patients. To our knowledge, there is not a lot of data available on possible gender-associated differences in the number of Th17 cells and CD4+ Tregs. One study on patients suffering from acute myeloid leukemia found a higher number of circulating Th17 in male healthy controls compared to females (54), another study on healthy adults found higher CD4+ Treg numbers in males compared to females (55); both studies support our findings in trauma patients, where males had both higher Th17 cell and CD4+ Treg percentages. The higher platelet count in patients with a lower ISS we observed might be attributable to less hemorrhaging—with subsequent loss of cellular blood components in particular—occurring in patients with fewer injuries. Furthermore, the platelet MCF was also higher in patients with lower ISS, which led us to the assumption that the previously described post-traumatic platelet dysfunction might be positively correlated to trauma severity. The results of the platelet MCF comparison between age groups was unexpected: it was actually lower in younger compared to older patients, which could raise the suspicion that younger patients might be more prone to the development of a platelet dysfunction. On the other hand, a shift toward a more procoagulant state is generally agreed upon for the elderly population, leading to a higher risk for thrombotic complications (56). This development is not exclusively, but at least in part, caused by the platelets' increased activity, which might just be what we observed here.

**Figure 5:**
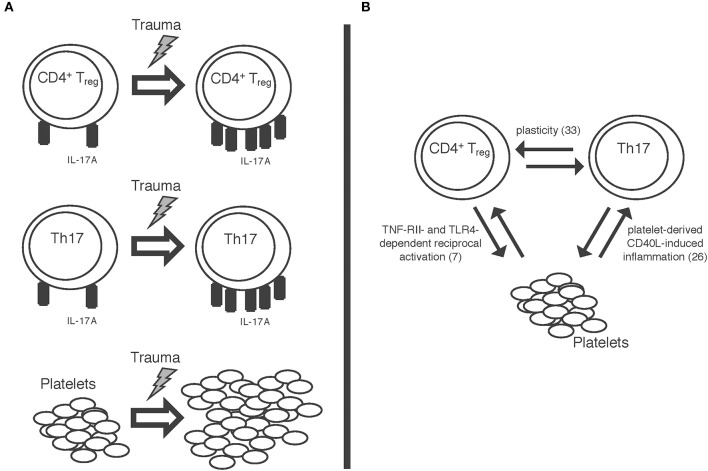
Schematic display of **(A)** the trauma-associated changes we were able to detect: increase in IL-17A expression on both Th17 cells and CD4+ regulatory T cells as well as increase in platelet count. **(B)** Some of the mechanisms possibly involved in these proceedings: a plasticity between CD4+ regulatory T cells and Th17 cells (33); reciprocal activation of CD4+ Tregs and platelets via TNF-RII- and TLR-4 dependent pathways (7); platelet derived sCD40L-dependent inflammation (26).

A schematic summary of some of our results is also displayed in Figure 5A. Trauma seems to impact both Th17 cells and CD4+ Tregs as well as platelets. The mechanisms of this and the potential interaction between these cell types remain unclear at this point; nevertheless, quite some interesting findings have been published by other workgroups, all of which might offer starting points for further studies in this field. For once, a significant plasticity between CD4+ Tregs and Th17 cells has been shown (33), meaning that T cells can convert into one another; furthermore, platelets have been shown to induce inflammation through sCD40L (26). In previous work from our group, we were able to show a tumor necrosis factor receptor 2- and toll-like receptor 4- dependent activation of CD4+ Tregs by platelets in a murine burn model (7) (Figure 5B). Taken together, while these findings cannot yet explain all the post-traumatic proceedings we discovered, these pathways should certainly be studied further in order to better understand the post-traumatic immunologic reaction.

Limiting factors of this present patient study include the solely descriptive and therefore non-interventional design, meaning that unlike in most animal models, no causalities can be proven. Furthermore, only 20 multiple trauma patients have been included to date, resulting in relatively small subgroups in our subgroup analysis. Therefore, we have not been able to correlate our findings with the patients' outcomes yet. Additionally, we have not compared our findings in the post-traumatic immune response with healthy controls thus far. These issues will, however, be addressed by enrolling more trauma patients and recruiting healthy volunteers, which will allow for a more thorough analysis of our results and subgroups. Another point to take into consideration might be the lack of a specific set of surface markers for the unambiguous identification of Th17 cells to date. We chose the co-expression of CD4, CD161, and CD196 as our identifying set for Th17 cells, as this combination has been used in a number of studies with satisfying results (24, 57). Furthermore, we have not taken blood transfusions into account. Considering platelet storage lesions have been proven to take place, their exact implications albeit remaining to be fully understood, especially the platelet function could be influenced and altered by transfusions (49). Lastly, infection and autoimmune diseases may influence both the count of Th17 cells and CD4+ Tregs as well as their IL-17A expression, we did, however, not factor those in as potential confounders.

In conclusion, this present study is the first to characterize IL-17A expression on peripheral blood Th17 cells and CD4+ Tregs of multiple trauma patients. We were able to demonstrate that these cell types actually increase their IL-17A expression during the first 10 days following trauma. The discovery of a subset of CD4+ Tregs expressing IL-17A following trauma suggests that their role in the post-traumatic immune response is not only an anti-inflammatory one, CD4+ Tregs might show just the same plasticity that has been described in other settings. Regarding platelets and hemostatic function in multiple trauma patients, our study shows that despite an increase in the absolute number of platelets in the trauma patients' blood over time, their function as measured thromboelastometrically actually decreases. These results support the notion of a platelet dysfunction occurring after trauma. Lastly, we were able to identify gender, age, and trauma severity as factors that influence and alter the analyzed parameters to various extents. Our data thereby adds to understand the hosts' susceptibility to trauma in dependence of gender, age, and trauma severity.

## Data Availability Statement

All datasets generated for this study are included in the manuscript/supplementary files.

## Ethics Statement

This study was carried out in accordance with the recommendations of the review committee of the Technical University of Munich with written informed consent from all subjects. All subjects gave written informed consent in accordance with the Declaration of Helsinki. The protocol was approved by the review committee of the Technical University of Munich (reference number 5925/13).

## Author Contributions

FH and MH conceptualized the study, designed the experiments, established the sample collection, processing protocol, analyzed and interpreted the data, and wrote the manuscript. FH, AD, and NK performed the experiments. MH, PB, MG, CC, and SH-W provided critical resources. FH, CC, SH-W, MG, and MH edited the manuscript. MH supervised the work.

### Conflict of Interest

This report includes experimental work performed by FH in fulfillment of her doctoral thesis requirements. The remaining authors declare that the research was conducted in the absence of any commercial or financial relationships that could be construed as a potential conflict of interest.

## Abbreviations

95% CI: 95% confidence interval
APC: Allophycocyanin
B: Regression coefficient
EDTA: ethylenediaminetetraacetic acid
GEE: Generalized estimating equations
IL-17A: Interleukin 17A
ISS: Injury severity score
MCF: Maximum clot firmness
MFI: Median fluorescence intensity
*p*: Probability value
PE: Phycoerythrin
SE: Standard error
Th17: T helper 17
CD4+ Treg: CD4+ regulatory T cell.
